# From raw data to actionable insights: preprocessing real-world data for machine learning in diabetes care

**DOI:** 10.3389/fdgth.2026.1685842

**Published:** 2026-02-25

**Authors:** Marco Montagna, Aleksandar Svilenov Rabadzhiev, Alberto Traverso, Emanuela Setola, Edoardo Draetta, Alessio Dimonte, Simone Barbieri, Bruno Fabiani, Lorenzo Piemonti, Antonio Esposito, Carlo Tacchetti, Patrizia Rovere Querini

**Affiliations:** 1 Department of Medicine, IRCCS San Raffaele Scientific Institute, Milan, Italy; 2 School of Medicine, Vita-Salute San Raffaele University, Milan, Italy; 3 S-RACE (S-Raffaele Ai CEnter), Vita-Salute San Raffaele University, Milan, Italy; 4 Diabetology Unit, IRCCS San Raffaele Scientific Institute, Milan, Italy; 5 Diabetes Research Institute, IRCCS San Raffaele Scientific Institute, Milan, Italy; 6 Experimental Imaging Center, IRCCS San Raffaele Scientific Institute, Milan, Italy

**Keywords:** explainability, glycated hemoglobin, machine learning, preprocessing pipeline, real world data, type 2 diabetes mellitus, predictive modeling

## Abstract

**Background:**

Machine Learning (ML) applied to healthcare Real World Data (RWD) may improve patient management. RWD, however, requires extensive preprocessing to make it ML-ready. Our aim was to explore the impact of preprocessing on ML models applied to RWD from 20 years of type 2 diabetes patients visits.

**Methods:**

Our cohort consisted of patients with at least two glycated hemoglobin (HbA1c) measurements three years apart. We set up three different experimental settings consisting of different data preprocessing pipelines. Logistic Regression (LR), XGBoost and a Decision Tree Classifier (DTC) were then applied and tuned to optimize precision.

**Results:**

The final dataset comprised 12 variables from 1,651 patients treated between 2003 and 2023. 921 (56%) patients had a HbA1c decrease at three years. This group had a higher baseline HbA1c, higher BMI and shorter first visit gap from the date of diagnosis (*p* < 0.0001). Precision scores for LR, XGBoost did not vary across different experimental conditions while DTC benefitted from missing data imputation. Shapley Additive Explanations confirmed the Exploratory Data Analysis findings, with worse baseline values being predictors of HbA1c decrease at three years.

**Conclusions:**

ML models' performance and their explanation did not vary substantially across experimental conditions, with worse baseline values being predictors of HbA1c decrease at three years. Insights such as this, extracted by ML application to RWD, enable clinical discussion and may foster improvements in patient management.

## 1 Introduction

Type 2 Diabetes Mellitus (T2DM) is a chronic disease and a growing health emergency, affecting approximately 10% of people worldwide. It is characterized by a high morbidity and mortality due to its complications (1). Serum glycated hemoglobin (HbA1c) is used in clinical practice as a biomarker for T2DM control in response to therapy and lifestyle changes. HbA1c is related to the average glucose blood levels in the past 3 months and can be directly linked to the risk of developing complications in patients with T2DM (2). HbA1c temporal evolution is affected by disease severity (e.g., insulin resistance, residual pancreatic function), lifestyle (e.g., diet, exercise) but, more importantly, by adherence to medications (3). However, most T2DM patients have complex clinical pictures composed of coexisting diseases, multiple undergoing therapies, ageing and socio-economical frailty. Therefore, treating teams find it difficult to anticipate disease trajectories of their patients and provide proactive rather than reactive treatments. Machine Learning (ML) is being extensively explored as a new tool to leverage Real World Data (RWD) generated in diabetes clinics (4–6). Attempts have been made in the prediction of disease onset, development of complications, HbA1c control and weight control (7–12). These studies explore the potential of ML in healthcare, particularly as data collection techniques rapidly advance and Electronic Health Records (EHRs) are increasingly used worldwide allowing to extract RWD and enable more precise disease management through computer-driven methodologies. However, key challenges associated with implementing ML in clinical settings are widely recognized (13–16). First and foremost, the influence of data preprocessing and RWD quality on the subsequent model training phase must be better elucidated (17–19).

Therefore, this methodological case study aims to provide insights into preparing real-world clinical data for application with different ML classification models. The primary focus was to thoroughly analyze the performance of these models across different experimental conditions and explore how data scientists and clinicians can collaborate to take optimal preprocessing decisions for improved outcomes. As a prototypical task, we chose the classification of patients into two classes, either decrease or increase in HbA1c at three years from the first available record in the EHR.

## 2 Materials and methods

### 2.1 Dataset

The dataset represents RWD collected and stored in the EHR (Smart Digital Clinic, METEDA S.r.l.) during routine clinical activity at the diabetes outpatient clinics of IRCCS San Raffaele Scientific Institute. Smart Digital Clinic is the last version of the EHR that is used in many Italian diabetes clinics allowing for structured and standardized data entry and nation-wide analyses (20). The EHR features a direct communication with the hospital's patients registry software: demographic data (age and sex) are therefore automatically imported and available for all encountered subjects. The EHR also follows ICD9 coding for diseases. Measurements are restricted by predefined categories that set their name and units, but the value field has no range restriction. This does not avoid data entry mistakes in the measurements that will require specific management.

Collection spans from 2003 to 2023. In May 2024 EHR data was exported in 11 separate.csv files and uploaded to the San Raffaele Artificial Intelligence Center of Excellence (S-RACE) platform for anonymization. S-RACE is a multimodal data integration cloud-based multilayer platform deployed at IRCCS San Raffaele Scientific Institute (21).

The collected data includes demographic information, clinical measurements, laboratory tests, treatment details, and other diagnostic results of patients diagnosed with T2DM.

This retrospective study was conducted in accordance with the pre-established regulations regarding data privacy, as described in the observational clinical study “AI-TRYDIA”, notified and approved by the Institutional Ethics Committee. According to the approved study protocol, no informed consent was required from patients as data was fully anonymized.

### 2.2 Cohort selection

**Figure 1:**
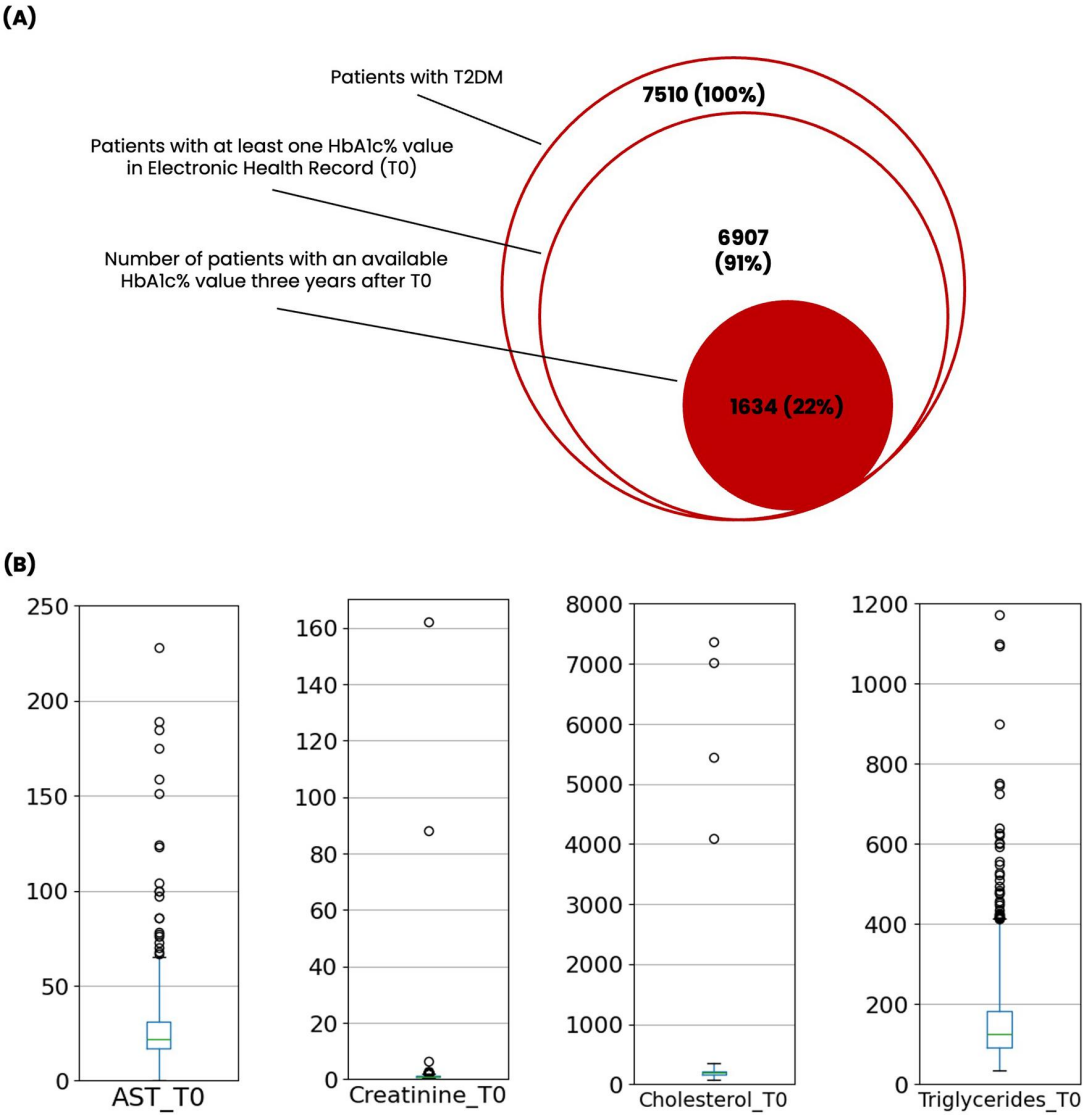
**(A)** Euler diagram illustrating the cohort selection process and relative dataset sizes. **(B)** Box plots showing the distribution of selected variables [Aspartate Amino Transferase (AST), Creatinine, Total Cholesterol, Triglycerides] at baseline, before data preprocessing. Whiskers cover the upper and lower fences that were mathematically determined for outlier identification as described in the materials and methods section (Q1-2.5*IQR and above Q3 + 2.5*IQR). Circles represent outlier measurements.

The study was conducted on individuals identified in the EHR as having a diagnosis of T2DM. The analysis included only patients having a first record of HbA1c (T0) and a second one after three years, within a range of ±180 days (T1). Additionally, patients with an initial HbA1c < 6.5% recorded prior to their T2DM diagnosis date were excluded from the final cohort, as they were considered non-diabetic at the time of their first HbA1c determination. A flowchart illustrating the cohort selection is presented in Figure 1A.

### 2.3 Exploratory data analysis

Exploratory Data Analysis (EDA) was performed before and after data processing to identify data entry errors, quantify missing variables, and uncover relationships between explanatory and outcome variables in a subgroup analysis. We employed descriptive statistics, including mean, median, minimum/maximum value, standard deviation, and interquartile range (IQR), along with visual tools such as histograms and box plots. To assess the significance of variables' distribution between subgroups, we used the chi-square test for categorical variables and the Mann–Whitney *U* test for the numerical variables, having verified their non-normal distribution via the Shapiro–Wilk test.

### 2.4 Data preprocessing

A preliminary point-wise data correction process consisted in manually repairing obvious data entry errors and duplicated records. Then, we applied a semi-automated outlier detection: clinicians of the team reviewed the upper and lower fences that were mathematically determined (below Q1-2.5*IQR and above Q3 + 2.5*IQR) to be appropriate for features that are naturally extremely skewed. The outliers were turned into NaN (Not a Number) values.

For numerical variables, we performed data format adaptation by replacing commas with dots and rounded to two decimal places to improve readability. Sex was the only categorical variable selected for model training; as a binary variable, we label encoded it (0/1). We calculated missing LDL cholesterol values by using Friedewald formula when the total cholesterol, HDL cholesterol and triglycerides values were present (22). Data standardization was performed, specifically for the logistic regression model, by using the StandardScaler function by sklearn with all the parameters set to default. This method transforms the features to have zero mean and unit variance, ensuring that all variables contribute equally to the logistic regression model and improving numerical stability during optimization.

### 2.5 Feature selection and engineering

Only a subset of available data was selected for the purpose of this study. We did not include medication, imaging and complications data but focused on demographic, anamnestic, anthropometric and laboratory data. Together with the engineered features, they were utilized to feed the predictive model. Predictive features were selected based on medical expertise and completeness in the dataset. For each patient, predictive laboratory features were censored on the same date as the HbA1c baseline measurement. Further measurements happening in the time window between T0 and T1 were not included in the analysis. Predictive features with >50% of missing data were removed after data pre-processing as they can negatively impact the accuracy and reliability of the model (Supplementary Figure S1). In addressing collinearity, we chose to remove only the weight feature, which has a high correlation with BMI (0.8), as indicated by Spearman's Correlation Coefficient (Supplementary Figure S2). Although total cholesterol and LDL cholesterol are highly correlated (0.91), we decided to retain both features because they offer distinct clinical insights.

As for the classification target, a patient's outcome was encoded as “True” if they experienced a decrease in HbA1c relative to baseline, and “False” otherwise. Additional engineered features were the age of patients at T0 and the “first visit gap”, i.e., the amount of time in years between the date of diagnosis of T2DM and T0.

### 2.6 Model choice, training and explanation

Our aim was the identification of patients who would experience a decrease in baseline HbA1c. We considered a small set of widely adopted classification models, balancing model explainability against model performance. We selected Logistic Regression (LR) and Decision Tree (DT) for their simplicity and explainability, and XGBoost as a less explainable model characterized by high generalization performance.

We k-fold cross-validated models using scikit-learn's StratifiedShuffleSplit, with a 70-30 split and stratification on the outcome variable. This method ensures that each fold of the cross-validation process maintains the same outcome distribution as the original dataset while randomly shuffling the data, reducing variability and improving the robustness of model performance estimates.

We evaluated models' performances across different experimental conditions with a set of metrics for classification from the sklearn.metrics library: Accuracy, Precision, Recall, F1 Score, Area Under the Receiver Operating Characteristic Curve (ROC AUC) score, and the Matthews Correlation Coefficient (MCC). We selected precision as the metric to be optimized (see Discussion). Hyperparameter settings for the three models can be found in the Supplementary Materials.

As a last step, we employed Feature Importance graphs and Shapley Additive Explanation Values (SHAP) to help understand the decision-making process of each model (23, 24). SHAP was recently employed in other studies in the context of either diabetes (25, 26) or pre-diabetes risk prediction (27).

### 2.7 Experimental conditions

Each model was tested in three distinct experimental settings: (i) all rows with missing values were removed from both the training and test sets; (ii) missing values in the training and test sets were separately replaced with the median of each corresponding predictive feature using the SimpleImputer module; (iii) missing data in the training and test sets was separately imputed using the Multivariate Imputation by Chained Equations (MICE) algorithm. SimpleImputer is a straightforward method that imputes missing values by replacing them with a fixed statistic for each feature while MICE generates imputed values by drawing from estimated conditional distributions of each variable given all others (28). To enhance the imputation technique of the MICE algorithm, the maximum number of iterations was increased to 50, the stopping condition tolerance was set to 0.01, and the minimum possible imputed value was set to 0.1 to ensure only non-negative values were imputed for the predictive features. Furthermore, the imputation strategy for the MICE imputer was set to “median” as it is less prone to outliers. Additionally, the XGBoost classifier was used with training and test sets containing missing values, as it can internally handle them.

### 2.8 Software and tools

Data analysis was performed in Visual Code Studio by using the computer programming language Python (version 3.11.5). Libraries such as pandas, NumPy, matplotlib, seaborn, and scikit-learn were installed and imported into a virtual environment to conduct analysis on textual and numeric data and to obtain visual representations such as graphs and bar plots. The same libraries were utilized to perform ML classification.

We qualitatively assessed the methodological soundness of our contribution and the reproducibility of its results according to the IJMEDI checklist for assessment of medical artificial intelligence, directly inspired by the MINIMAR guideline (see Supplementary Materials) (29, 30).

## 3 Results

### 3.1 Raw data characteristics

The original RWD extraction contained a total of 7,510 patients. After applying the cohort selection criteria, the final dataset consisted of 1,634 patients, that is 21.8% of the available starting population (Figure 1A).

Table 1 shows the summaries of predictor variables before data pre-processing. The total number of available records for the predictive variables ranges from 119 to 1,634, indicating a very diverse missingness pattern along variables. Of note are the evident outlier values for some of the variables, possibly result of data entry mistakes during the outpatient visits. For instance, a value of 0 for laboratory features such as creatinine and AST are unplausible, as well as a maximum value of 7,375 mg/dL, and 1,172 mg/dL for total cholesterol and triglycerides, respectively (Figure 1B).

**Table 1:** Descriptive statistics of the dataset before data processing.

Variable	Count	Mean (Std Dev)	Median (Q1–Q3)	Min	Max
Age T0 (years)	1,634	63.71 (11.5)	64.00 (56–71)	18.00	97.00
BMI T0 (Kg/m²)	1,299	28.95 (11.9)	27.90 (24.9–31.3)	17.10	307.60
Cholesterol HDL T0 (mg/dL)	1,002	49.69 (14.6)	47.00 (40–57)	23.00	177.00
Cholesterol LDL T0 (mg/dL)	554	108.10 (35.9)	108.00 (82–132)	17.40	235.00
Creatinine T0 (mg/dL)	767	1.30 (6.6)	0.90 (0.8–1.1)	0.00	162.00
Diastolic Blood Pressure T0 (mmHg)	1,309	75.86 (10.4)	80.00 (70–80)	44.00	155.00
Fasting Blood Sugar T0 (mg/dL)	1,411	155.81 (56.3)	142.00 (122–173)	1.00	548.00
First Visit Gap (years)	1,634	9.68 (15.8)	6.00 (1–12)	−15.00	114.00
ALT T0 (UI/L)	404	36.30 (36.9)	26.00 (18–40)	0.00	435.00
HbA1c T0 (%)	1,634	7.61 (1.7)	7.20 (6.5–8.3)	2.70	16.60
HbA1c T1 (%)	1,634	7.08 (1.1)	6.90 (6.4–7.6)	2.97	14.20
Number of comorbidities	119	1.79 (1.0)	1.00 (1–2)	1.00	6.00
AST T0 (UI/L)	437	28.87 (24.6)	22.00 (17–31)	0.00	228.00
Systolic Blood Pressure T0 (mmHg)	1,315	134.82 (18.5)	135.00 (120–146)	7.00	235.00
Total Cholesterol T0 (mg/dL)	1,120	212.82 (357.2)	191.00 (160–222)	76.00	7,375.00
Triglycerides T0 (mg/dL)	1,025	157.14 (112.7)	126.00 (91–183)	35.00	1,172.00
Weight T0 (kg)	1,459	78.80 (25.0)	76.00 (67–88)	39.00	800.00

### 3.2 Processed data characteristics and subgroup analysis

Table 2 shows descriptive statistics of the dataset after data processing. A total of 13 predictive variables were chosen to be fed into the predictive models. At the time of their first recorded measurement following a T2DM diagnosis, patients had a median age of 64 years, with 38% being females.

**Table 2:** Descriptive statistics of the dataset after data processing.

Variable	Count	Mean (Std Dev)	Median (Q1–Q3)	Min	Max
Age T0 (years)	1,633	63.74 (11.46)	64.00 (56–71)	20.00	97.00
BMI T0 (Kg/m²)	1,291	28.43 (5.01)	27.80 (24.9–31.3)	17.10	46.50
Cholesterol HDL T0 (mg/dL)	994	49.11 (12.91)	47.00 (40–56)	23.00	96.00
Cholesterol LDL T0 (mg/dL)	893	111.71 (36.67)	110.40 (85.8–135.8)	17.40	244.80
Diastolic Blood Pressure T0 (mmHg)	1,303	75.69 (9.83)	80.00 (70–80)	45.00	105.00
Fasting Blood Sugar T0 (mg/dL)	1,369	149.77 (44.54)	141.00 (121–169)	1.00	300.00
First Visit Gap (years)	1,558	7.90 (8.33)	5.00 (1–12)	0.00	39.00
Females	1,634	38%			
HbA1c T0 (%)	1,603	7.49 (1.47)	7.10 (6.5–8.2)	2.70	12.80
Systolic Blood Pressure T0 (mmHg)	1,311	134.84 (17.62)	135.00 (120.0–145.5)	60.00	191.00
Total Cholesterol T0 (mg/dL)	1,116	192.14 (44.01)	191.00 (159–221)	76.00	359.00
Triglycerides T0 (mg/dL)	990	142.15 (71.57)	124.50 (90.0–174.8)	35.00	413.00

Reported results are on the dataset without the application of data imputation techniques.

Subgroup analysis revealed a good balance in terms of distribution of the outcome, with the HbA1c increase group comprising 713 patients (44%) and the decrease group comprising 921 patients (56%) of the total cohort. Interestingly, most laboratory predictive features for the decrease group had higher baseline values compared to the increase group. No statistically significant difference was noted between the two groups in terms of age and gender distribution (Table 3).

**Table 3:** Descriptive statistics for the increase and decrease groups. Categorical variables are expressed as count (percentage) and continuous variables as median (interquartile range).

Variable	Increase Group (*n* = 713)	Decrease Group (*n* = 921)	*P*-value
Age T0 (years)	65.00 (56–71)	64.00 (56.0–71.3)	0.98
BMI T0 (Kg/m²)	27.40 (24.5–30.6)	28.20 (25.2–31.8)	<0.0001
Cholesterol HDL T0 (mg/dL)	49.00 (41–58)	46.00 (39–55)	<0.0001
Cholesterol LDL T0 (mg/dL)	109.40 (84.7–134.7)	110.80 (86.9–137.6)	<0.0001
Diastolic Blood Pressure T0 (mmHg)	75.00 (70–80)	80.00 (70–80)	<0.0001
Fasting Blood Sugar T0 (mg/dL)	132.00 (115–153)	150.00 (127.8–185.3)	<0.0001
Females	277 (38.8)	337 (36.6)	0.38
First Visit Gap (years)	6.00 (2–13)	5.00 (0–11)	0.0002
HbA1c T0 (%)	6.60 (6.1–7.2)	7.80 (7–9)	<0.0001
Systolic Blood Pressure T0 (mmHg)	130.00 (120–145)	136.00 (125–150)	<0.0001
Total Cholesterol T0 (mg/dL)	187.00 (156–216)	193.00 (163–225)	<0.0001
Triglycerides (mg/dL)	115.00 (84–166)	132.00 (97–185)	<0.0001

**Figure 2:**
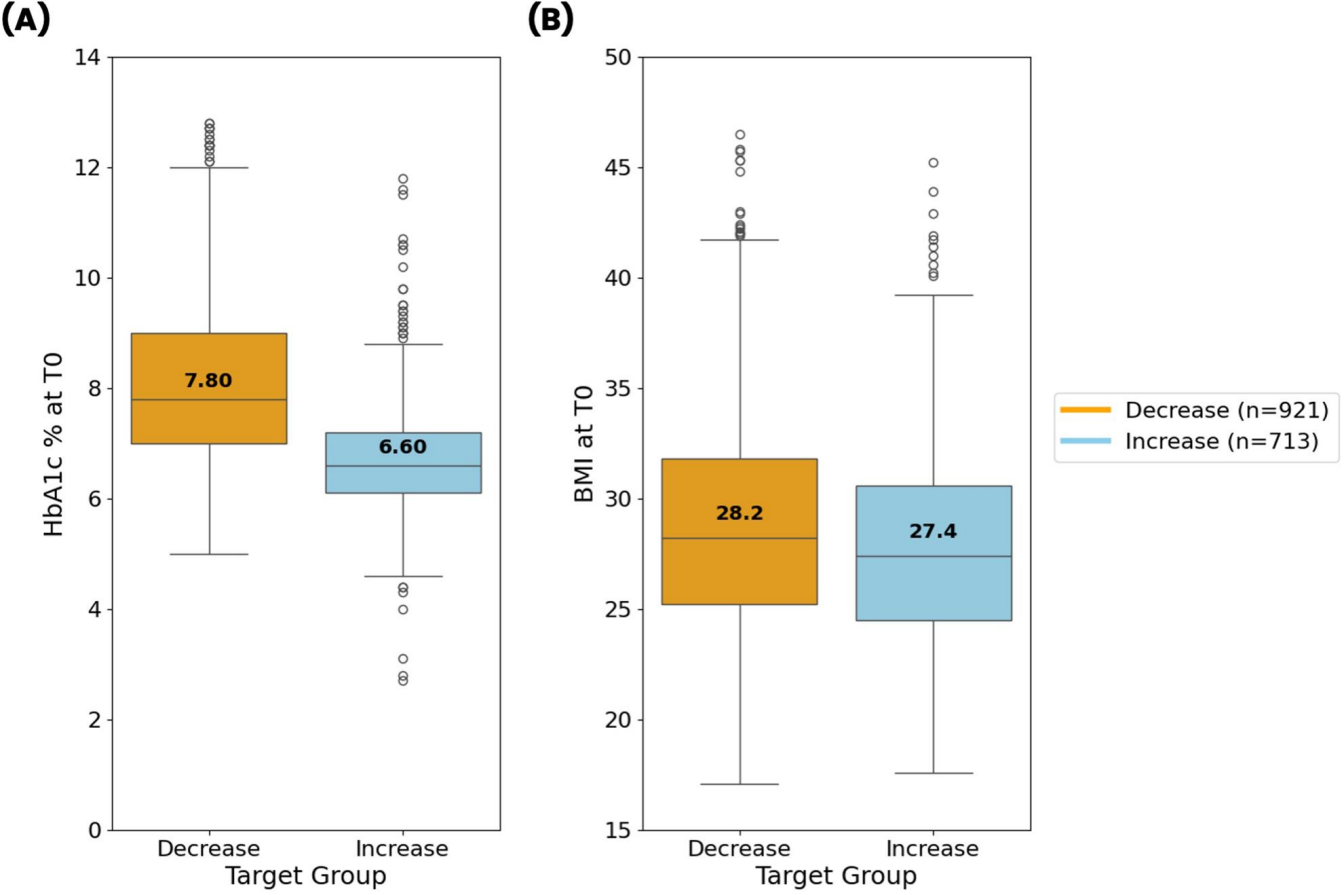
Comparison of **(A)** baseline glycated hemoglobin (HbA1c) and **(B)** baseline BMI levels between decrease (orange) and increase groups (light blue) after data preprocessing. Median values for each group are reported over the corresponding line in the box plots.

A comparison of baseline levels of HbA1c and BMI for the decrease and increase groups is shown in Figures 2A,B. In the decrease group, the median values for HbA1c and BMI are elevated at 7.8 and 28.2, respectively, compared to the increase group, which has median values of 6.6 for HbA1c and 27.4 for BMI.

### 3.3 ML models predictive performance and explanation

**Figure 3:**
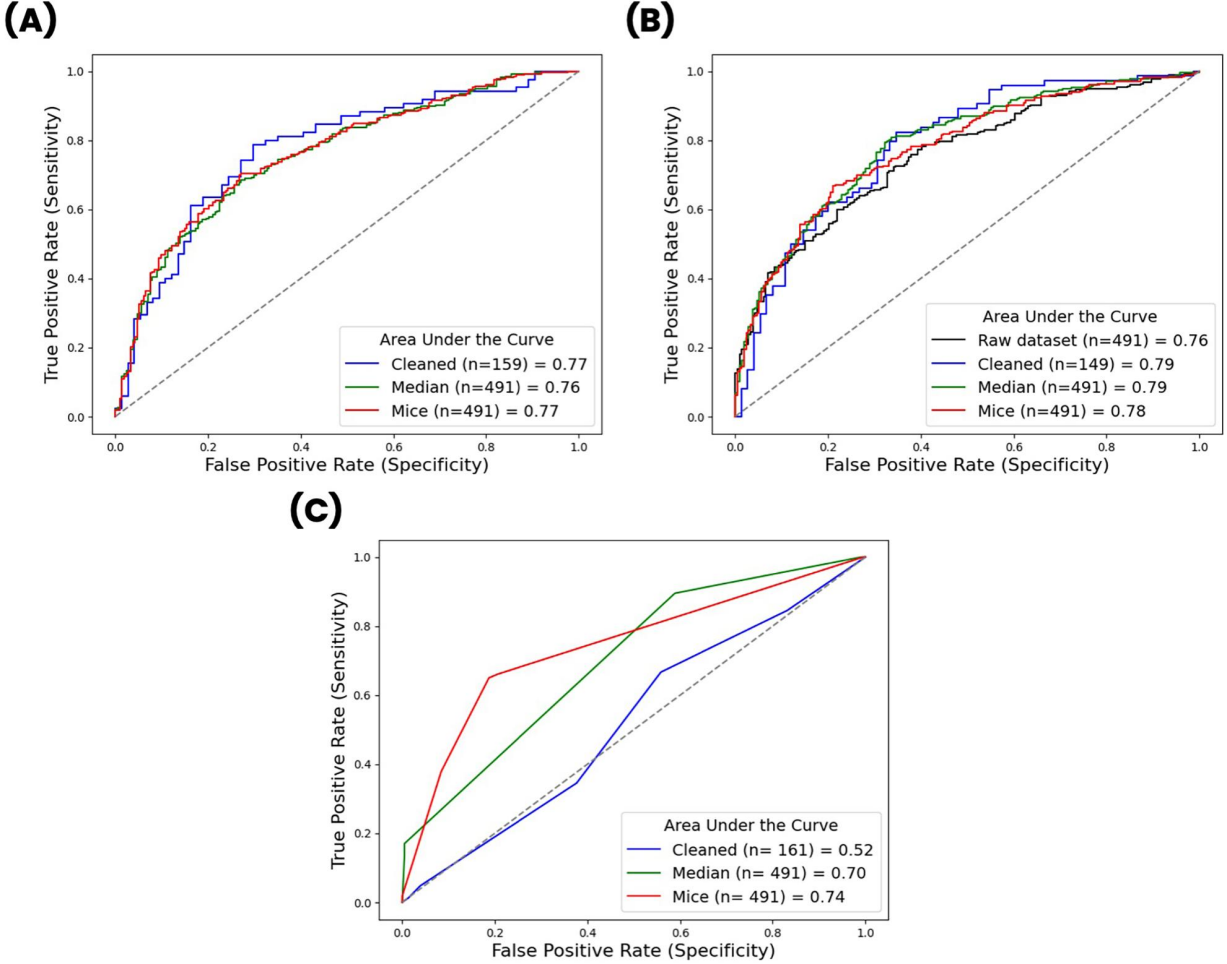
Receiver operator characteristic curves of **(A)** logistic regression models, **(B)** XGBoost models and **(C)** decision tree classifier models across different data preprocessing conditions. Dataset sizes for each condition are reported in the legend together with the corresponding area under the curve.

Table 4 shows the generalized predictive performance of the three models when trained on datasets where missing values had been differently managed. Accuracy, precision, recall, F1 score and the Matthews Correlation Coefficient for each condition are reported in Supplementary Tables S1–S3. ROC curves are reported in Figure 3. We checked the distribution of variables imputed with the MICE method. Consistently with the strategy of imputation being set to “median” (see Section 2.7), the imputed variables show an increased density around the median of the distribution (Supplementary Figure S3), but no out-of-distribution values were generated.

**Table 4:** Precision scores of the three models across different experimental conditions. Average results across all five folds are shown. Logistic regression and decision tree classifier performances when including missing values in the dataset could not be measured, as these models cannot manage them.

Experimental setting	Logistic Regression	XGboost	Decision Tree Classifier
Missing values not removed	N/A	0.74	N/A
Missing values removed	0.75	0.72	0.62
Missing values replaced with median value	0.76	0.74	0.70
Missing values imputed via MICE algorithm	0.77	0.74	0.77

The top-ranked features when applying model explanation algorithms were consistent across the three different models and experimental conditions, being HbA1c, age and BMI at baseline, and first visit gap (see Supplementary Figures S6–S11).

## 4 Discussion

Machine learning (ML) is becoming a key tool for analyzing large real-world datasets from healthcare centers, offering insights into disease trajectories in complex scenarios like Type 2 Diabetes Mellitus (T2DM) (31, 32). However, applying ML to RWD requires extensive data preprocessing, and standardized pipelines remain unavailable. The impact of preprocessing choices on model performance is often unclear and depend on the clinical context and questions. This methodological case study aimed to evaluate decision-making in ML pipelines and its impact on three prototypical classification models. We show that Logistic Regression and XGBoost models provide stable performances across experimental condition, while the Decision Tree Classifier benefits from data imputation. We also demonstrate that the adoption of the more computationally expensive Multivariate Imputation by Chained Equations compared to simple median imputation does not provide a performance advantage.

The original number of patients available is decent, but after applying the cohort selection criteria we lose 88% of our sample size (Figure 1A). Such data loss is to be expected when working with RWD for longitudinal studies such ours, as it is not uncommon for patients to have a single encounter with a treating center.

As seen in Table 1, raw RWD suffers from relevant mistakes in data entry and from great variety in the number of actual records available for each variable, according to how often and carefully they are measured and inputted in the busy everyday clinical activity. In this regard, there is an urgent need to increase data literacy of the clinical teams and put in place strategies to optimize data quality from the real beginning (33). Of note, key determinants of HbA1c trajectory were not included in model training. For example, medication adherence is not recorded in the EHR and was therefore not available in the RWD extraction. Also, comorbidities, albeit recorded in the EHR, were available for only 7% of the cohort and were therefore excluded in the variable selection step. Then, outliers must be then identified and excluded. We found that mathematical identification of the boundaries for outlier must be coupled with clinical oversight, in a continuous discussion between data scientists and clinicians allowing optimal decision-making for the least information loss possible.

The same can be stated for the selection of features and for possible feature-engineering strategies. In our case, clinical oversight allowed to increase LDL counts using a clinically validated formula, and overwrite feature exclusion decisions based on collinearity, due to the different informative content of the various components of total cholesterol. The choice of the target metric for optimization was also based on multidisciplinary discussion. From a clinical perspective, it was more important to prevent the model from misclassifying patients into the decrease group when they belonged in the increase group. For this reason, we prioritized optimizing the precision score, which measures the proportion of true positive predictions among all predicted positives.

At the subgroup analysis it is evident how patients in the HbA1c decrease group have significantly higher HbA1c, worse pressure and lipid control and higher BMI values at T0. We hypothesize that patients with such baseline clinical picture receive more aggressive treatment, leading to greater reductions in HbA1c over time. Additionally, patients in the decrease group have a median of 1 year less in the gap between diagnosis and first encounter at our center, suggesting that patients with longer disease histories are more resistant to treatment. Interestingly, HbA1c T0 and first visit gap come out as the most important features that explain the decisions our ML models, consistently with findings from the EDA, suggesting that our analyses are robust and able to detect actual behaviors hidden in the data.

Notably, each model demonstrated similar evaluation metrics scores, across these conditions, suggesting that the use of complex imputation strategies may not be justified. Such strategies can inadvertently introduce errors into the dataset, potentially compromising the robustness of the models. The only exception was the decision tree classifier, which showed significantly improved performance when missing values were replaced with the median or imputed using the MICE algorithm, compared to when removed. Furthermore, when using machine learning models like the XGBoost classifier, which can natively handle missing values, it may be more efficient to forgo data imputation altogether—especially if the model's performance remains unaffected. Given the aims of this study, we intentionally restricted the number of models to benchmark focusing on three widely used models in healthcare. Other classes of models exist, all designed to deliver a performance comparable to state-of-the-art ML methods like Random Forest and Boosted Trees, while being highly explainable. Examples are the Explainable Boosting Machines, part of Generalized Addittive Models (34), or newer approaches employing neural networks such as TabNet (35).

Our study can act as a scaffold upon which modifications can be made to optimize the usage of ML models in the healthcare setting. While our goal was not to develop a model ready for immediate implementation in healthcare to revolutionize patient treatment planning—acknowledging that this requires further steps—we offered physicians valuable insights into factors that contribute to long-term diabetes management and possible biases affecting their clinical activity. Ultimately, this could lead to incremental improvements in patient care and disease management.

### 4.1 Limitations

The study has some limitations. First, it is a single-center study, which may limit the generalizability of the findings. Therefore, as a next step we plan to perform the external validation on an additional dataset from another institution. Second, the models relied on a few features and did not include others that could significantly influence HbA1c changes such as treatment choices, medication adherence, comorbidities, and lifestyle habits. Future experiments will extend the number of included features and evaluate the effect of era-specific treatments on the outcome. However, the fact that the models retained good performance despite not incorporating treatment-era features suggests that the selected baseline variables (e.g., HbA1c_T0, BMI_T0) are robust predictors of long-term HbA1c changes. A third limitation lies in the 20-year period of data collection, during which updates were made to diabetes management guidelines, new antidiabetic drugs were introduced, and physicians' habits, such as their willingness to intensify therapy or adjust treatment plans over time, may have changed. Future research could benefit from focusing on more recent data to provide an up-to-date, nuanced understanding of evolving treatment practices and their impact on long-term diabetes management. Finally, the adopted cohort selection strategy may introduce survivorship bias, as it inherently excludes patients who lacked sufficient follow-up data to compute the target variable (HbA1c change at 3 years). However, the resulting cohort retained a representative proportion of “failures”, with 44% of patients experiencing HbA1c worsening.

### 4.2 Conclusions

Across varying experimental conditions, precision scores for logistic regression and XGBoost models remained stable, whereas decision tree classifiers showed improved performance following missing data imputation. SHAP analyses corroborated insights from exploratory data analysis, reinforcing the robustness of feature importance across models. Overall, machine learning model performance and interpretability remained consistent, highlighting that poorer baseline clinical values were predictive of a significant HbA1c reduction over a three-year period. These findings demonstrate the potential of applying machine learning to real-world data for generating clinically meaningful insights, thereby supporting more informed discussions and strategic enhancements in patient management.

## Data Availability

The data that support the findings of this study are not publicly available due to privacy or ethical restrictions. Requests to access the datasets should be directed to montagna.marco@hsr.it. The code is publicly available at DOI: 10.17632/j26gdxcw7f.1.
